# Supplementary material for: Diversity of Neurotransmitter-Producing Human Skin Commensals
Source: Int J Mol Sci. 2024 Nov 17;25(22):12345. doi: 10.3390/ijms252212345 (PMC11595044; doi:10.3390/ijms252212345)
Supplement: Supplementary file 1 [file ijms-25-12345-s001.zip › Supplementary files.pdf]

Diversity of Neurotransmitter Producing Human Skin Commensals.

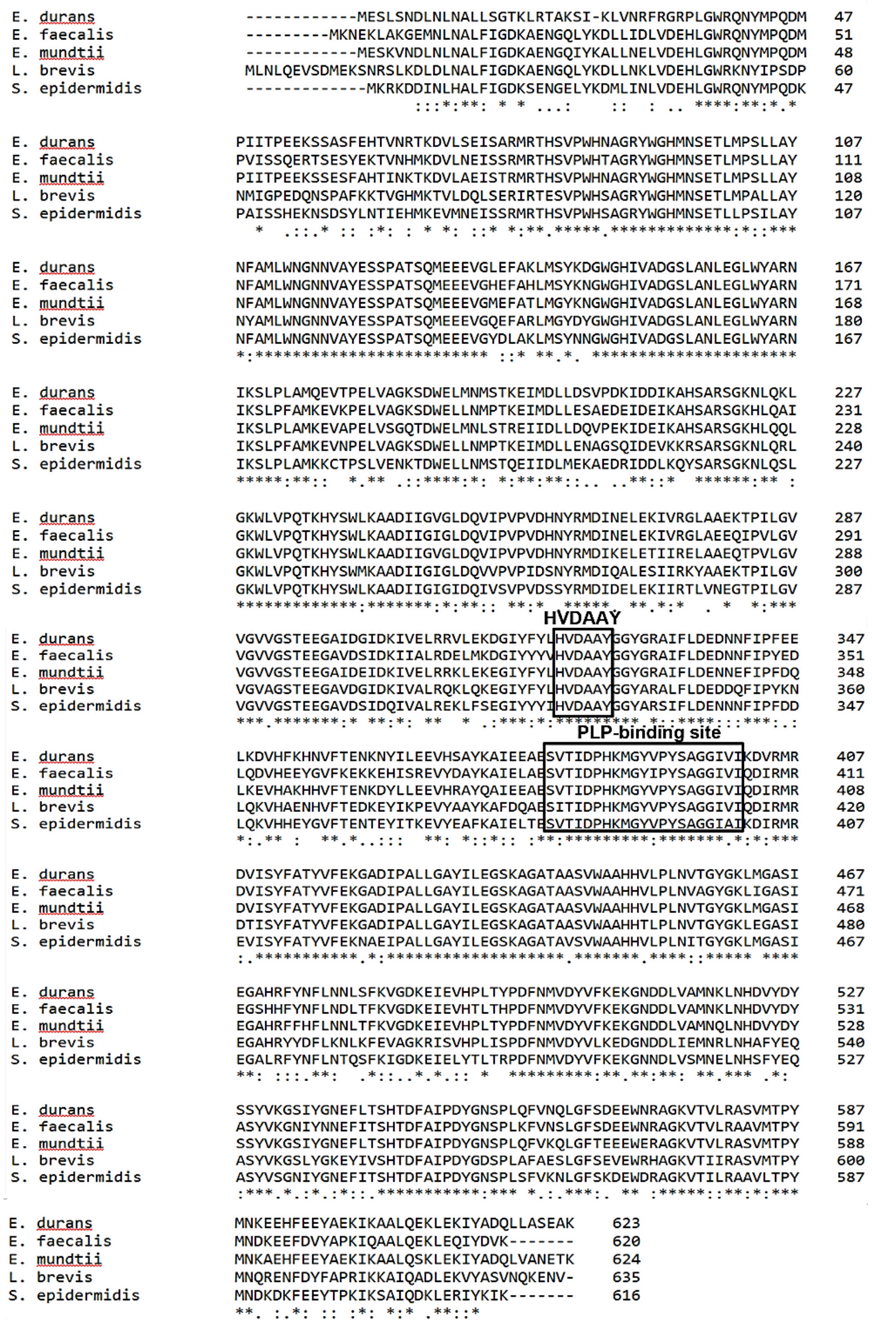

Figure S1: Comparison of the amino acid sequence of tyrosine decarboxylase from *S. epidermidis* 102 with those of *Enterococcus durans* IPLA 655 (GenBank accession no. CAF33980), *E. faecalis* JH2-2 (GenBank accession no. AAM46082), *E. mundtii* QU 25 (GenBank accession no. BAO05941.1), and *Levilactobacillus brevis* IOEB (GenBank accession no. AAN77279) strains using Clustal Omega (1.2.4). The conserved HVDAAY motif and the PLP binding site are boxed. Conserved residues are marked with an asterisk (*).

```
SadA      MEMEFNENNIDLETIIRDEV--NKYLSRDIGDLPATQQAPLELREKYEKMEVPN-----K  53
TDC       --MK-RKDDINLHALFIGDKSENGELYKDM--LINLVDEHLGWRQNYMPQDKPAISSHEK  55
            *: .:::*:*.::: .:    *  * :*:  *    :  *  *::*   : *      *

SadA      GRDIY----EVLKDLNNEVLNYLYR---PNH--------PRSFSFIP---GPASRLSWLG  95
TDC       NSDSYLNTIEHMKEVMNEISSRMRTHSVPWHSAGRYWGHMNSETLLPSILAYNFAMLWNG  115
          . * *    * :*:: **: . :     * *         .* :::*   .    : * *

SadA      DILTTANNIHASNFANATLPINIERNLINYLVGKIGYEIK------PAGGVFVSGGSMAN  149
TDC       NNVAYESSPAT-----------------SQMEEEVGYDLAKLMSYNNGWGHIVADGSLAN  158
          : ::  ..  :                 . :  ::**::        . * :*:.**:**

SadA      LTAIVAARDAQVE-------------------------------------MEDIKK---  168
TDC       LEGLWYARNIKSLPLAMKKCTPSLVENKTDWELLNMSTQEIIDLMEKAEDRIDDLKQYSA  218
          * .:  **: :                                       ::*:*:

SadA      --------ATVYLTSQT-HHSVGKALHVAGFLKNNIRRIEYNDDFTMNTDSLKAAIDKDI  219
TDC       RSGKNLQSLGKWLVPQTKHYSWLKAADIIGIGIDQIVSVPVDSSYRMDIDELEKIIRTLV  278
                     :*. ** *:*  ** .: *:  ::*  :  :..: *: *.*:  *  . :

SadA      EEGYKPAMVIATAGTTNTGSVDDFTTLGDIC-----DHYNLWLHVDGAYGLSH---I---  268
TDC       NEGTPILGVVGVVGSTEEGAVDSIDQIVALREKLFSEGIYYYIHVDAAYGGYARSIFLDE  338
          :**     *:...*:*: *:**.:  :  :      :    ::***.***      :

SadA      --------------------------LSDKAQHLFKGIERSDSASWDAHKLLFQTYSCAM  302
TDC       NNNFIPFDDLQKVHHEYGVFTENTEYITKEVYEAFKAIELTESVTIDPHKMGYVPYSAGG  398
                                    ::.:. . **.** ::*.: * **: :  **..

SadA      VIVKEKNHLLQSFGEDAEYLDDIASDDDVIDPEMLGIELT----RPARALKLWITLQVIG  358
TDC       IAIKDIRM-----REVISYFATYVFEKNAEIPALLGAYILEGSKAGATAVSVWAAHHVLP  453
          : :*: .       *  .*:   . :.:.  * :**  :       * *:.:* : :*:

SadA      EDEIIERIKYGEGLAEYAEEYVSSLDNWRI-----------------ISHANLSIVNFRY  401
TDC       LN----ITGYGKLMGASIEG---ALRFYNFLNTQSFKIGDKEIELYTLTRPDFNMVDYVF  506
           :       **: :.   *    :*  :.:                 ::: ::.:*:: :

SadA      ENSDLTEAQNNQLNSMAAQK---------IADSGYAIAYTTVLNNQRVIRLCTTNPLTTH  452
TDC       K-----EKGNNDLVSMNELNHSFYEQASYVSGNIYGNEFITSHTD-FAIPDYGNSPLSFV  560
          :     *  **:* **   :         ::.. *.  : *  .:  .*    ..**:

SadA      DDIRET--------------INRLDQYIGDYDMKDA--------------------      474
TDC       KNLGFSKDEWDRAGKVTILRAAVLTPYMNDKDKFEEYTPKIKSAIQDKLERIYKIK      616
          .::  :                 *  *:.* *   :
```

Figure S2: Comparison of the amino acid sequences of tyrosine decarboxylase from *S. epidermidis* 102 and SadA from *S. epidermidis* O47 (GenBank accession no. QKN61770.1) using Clustal Omega (1.2.4). Conserved residues are marked with an asterisk (*).

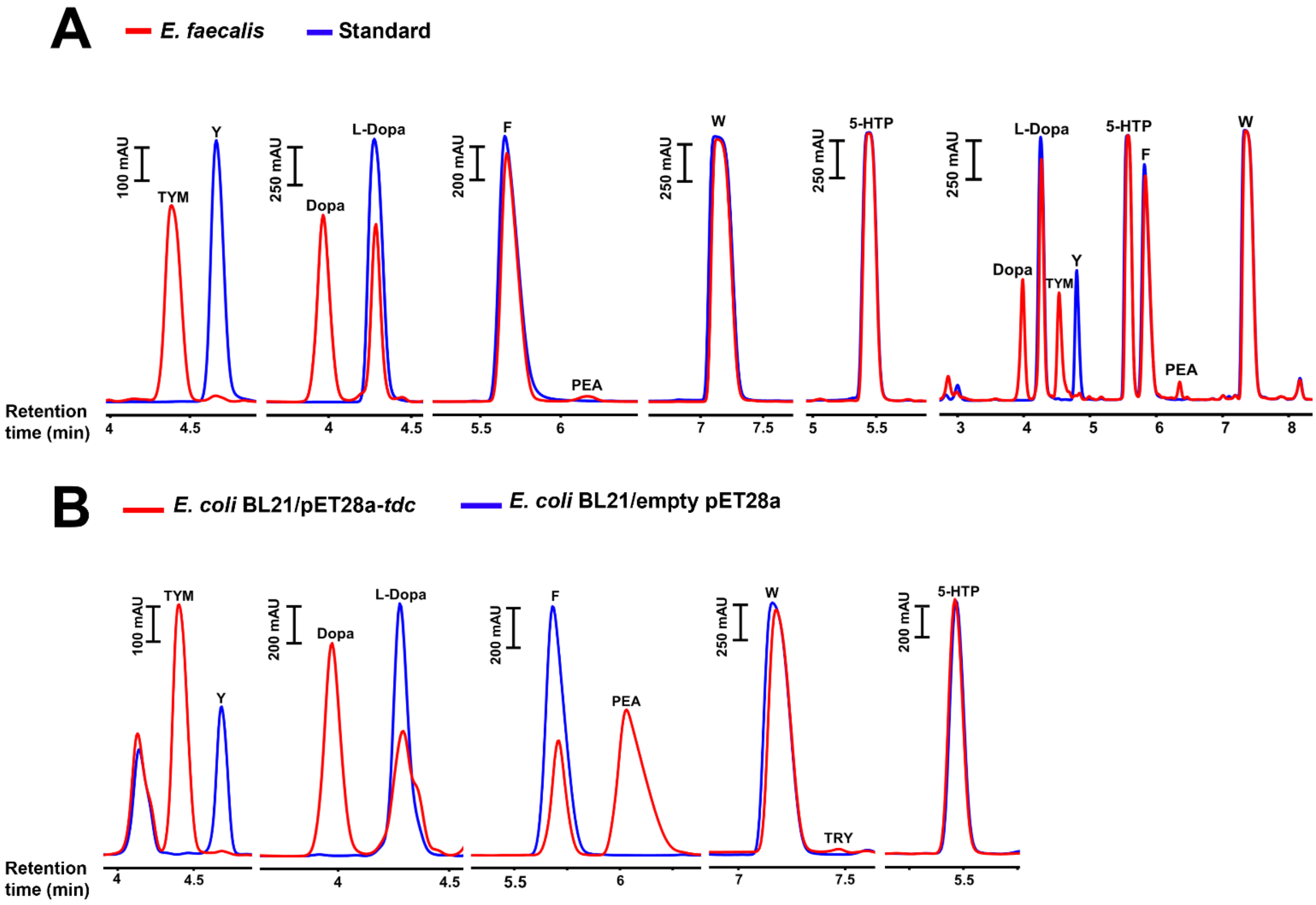

Figure S3: RP-HPLC chromatograms of neurotransmitter production by: **A)** *E. faecalis* ATCC 19433 after overnight incubation in 5xPBS buffer/10% glucose containing individual or a combination of substrates (1 mg/mL each). 5xPBS buffer/10% glucose supplemented with substrates (1 mg/mL each) was used as the standard; **B)** *E. coli* BL21 transformants harboring *tdc* gene from *E. faecalis* ATCC 19433 in the vector pET28a after overnight incubation in 5xPBS buffer/10% glucose containing individual substrates (1 mg/mL each) in comparison with transformants harboring empty plasmid. **5-HTP**, 5-hydroxytryptophan; **W**, tryptophan; **F**, phenylalanine; **Y**, tyrosine; **TRY**, tryptamine; **PEA**, phenethylamine; **Dopa**, dopamine; **TYM**, tyramine.

Table S1: Total bacterial count in the antecubital fossa area from 30 healthy subjects.

Volunteer	Age (years)	Gender	Total bacterial count (LogCFU/100 cm^2)
1	76	M	2.46
2	37	F	5.77
3	32	M	3.60
4	24	M	6.14
5	26	F	5.03
6	31	F	3.99
7	28	F	3.00
8	31	F	3.23
9	24	F	4.70
10	29	F	3.63
11	63	M	3.22
12	24	M	3.03
13	34	M	3.15
14	64	M	5.06
15	32	F	2.28
16	35	F	5.07
17	28	F	3.23
18	26	M	3.38
19	30	F	4.07
20	30	M	3.36
21	26	F	3.49
22	24	F	5.39
23	39	F	5.33
24	26	M	3.20
25	31	M	4.17
26	29	M	3.51
27	22	M	3.01
28	29	M	3.79
29	58	M	3.47
30	58	F	2.49

CFU, colony forming unit**; F,** female**; M,** male

Table S3: Primer sets for detection of sadA gene in *Staphylococcus* spp.

Primer set (5′→3′)	Length (bp)	Species	Strain	NCBI accession number
**F,** CKGATAAGATATGTGAWAGTCC<br>**R,** GTMTTTGTATCTGGYGGTTCWA	394	*S. aureus*	MS4	CP009828.1
			M013	CP003166.2
			RIVM6519	CP015173.1
			SA957	CP003603.1
		S. capitis	Sc1516941	CP145206.1
		S. caprae	JMUB145	AP018585.1
		S. carnosus	LTH 3730	CP016760.1
			TM300	AM295250.1
		S. condimenti	StO 2014-01	CP018776.1
			DSM 11674	CP015114.1
		S. epidermidis	O47	CP040883.1
			SEI	CP009046.1
			PM221	HG813242.1
		S. haemolyticus	JCSC1435	AP006716.1
		S. hominis	S00278	NZ_JACHLT010000017
			SDD3	VJMX01000002.1
		S. lugdunensis	RMLUG4	CP084440.1
		S. petrasii subsp. pragensis	CCM 8529	SRPJ01000003.1
		S. saprophyticus	sap-wu-085	JARZPO010000009.1
**F,** GCGTTACGTTGATACAGAGA<br>**R,** CCAGCACGATATGTATTTAGC	363	*S. coagulans*	M611545/16/1	JABTCP010000002.1
		S. schleiferi	TSCC54	AP014944.1
			1360-13	CP009470.1
			NCTC12218	LR962863.1
**F,** GGTGTTTTTGTTTCAGGCGGTTC<br>**R,** CCATAAGCGCCATCTGCATGGA	377	*S. lutrae*	ATCC 700373	CP020773.1

Table S7: Sequences of the oligonucleotides used in construction of *S. epidermidis* 102 mutant lacking *tdc* gene (102Δ*tdc*) and expression vectors.

**Primers**	**Sequence 5′→3′**
KOS1F	CGCGCAGATCTGTCGACGATAATTAAAACCCCTTTCTCTTTTTTC
KOS1R	GAGATGGATTTTTACCTCCAAAAGGTACAAACACTACTGAGC
KOS2F	CAGTAGTGTTTGTACCTTTTGGAGGTAAAAATCCATCTC
KOS2R	TGCAGGCATGCAAGCTTGATCTAAAATTAATTGTTGTTTTAATGAGG
KOS3F	GCGTTACGGGTATTAGCTTT
KOS3R	GGGGTGTTCATTTCCCTTAT
32S	GGTGGTGGTGCTCGAGTGCTTATTTTACGTCGTAAATTTGTTC
33S	CGCGGCAGCCATATGGCTAGCATGAAAAACGAAAAATTAGCAAAAG
42S	GCAGGAATATCGGCACCTTT
44S	GACACATCGTTGCTGATGGT
78S	GGTGGTGGTGCTCGAGTGCTTATTTAATTTTATAAATTCTTTCTAATTTATCTTG
79S	CGCGGCAGCCATATGGCTAGCATGAAAAGAAAAGACGATATCAATTTG
81S	GAAACAGCTGTAGCACCAGC
82S	GCATTATTCTTGGTTAAAAGC
